# Emergence of HIV-1 drug resistance mutations in mothers on treatment with a history of prophylaxis in Ghana

**DOI:** 10.1186/s12985-018-1051-2

**Published:** 2018-09-17

**Authors:** Alexander Martin-Odoom, Charles Addoquaye Brown, John Kofi Odoom, Evelyn Yayra Bonney, Nana Afia Asante Ntim, Elena Delgado, Margaret Lartey, Kwamena William Sagoe, Theophilus Adiku, William Kwabena Ampofo

**Affiliations:** 1 Department of Medical Laboratory Sciences, School of Biomedical & Allied Health Sciences, College of Health Sciences, University of Ghana, P.O. Box KB 143, Korle Bu, Accra, Ghana; 2 Department of Virology, Noguchi Memorial Institute for Medical Research, College of Health Sciences, University of Ghana, Legon, Accra Ghana; 3 Centro Nacional de Microbiología, Department Patogenia Viral/Uni of Biology and Variability of HIV, Instituto de Salud Carlos III, Ctra. de Majadahonda a Pozuelo, Km. 2, 28220 Majadahonda, Madrid Spain; 4 School of Medicine and Dentistry, College of Health Sciences, University of Ghana, Accra, Ghana

**Keywords:** Antiretroviral therapy, Phylogenetic analysis, Drug resistance profiles, Treatment outcome

## Abstract

**Background:**

Antiretrovirals have been available in Ghana since 2003 for HIV-1 positive pregnant women for prevention of mother-to-child transmission (PMTCT). Suboptimal responses to treatment observed post-PMTCT interventions necessitated the need to investigate the profile of viral mutations generated. This study investigated HIV-1 drug resistance profiles in mothers in selected centres in Ghana on treatment with a history of prophylaxis.

**Methods:**

Genotypic Drug Resistance Testing for HIV-1 was carried out. Subtyping was done by phylogenetic analysis and Stanford HIV Database programme was used for drug resistance analysis and interpretation. To compare the significance between the different groups and the emergence of drug resistance mutations, *p* values were used.

**Results:**

Participants who had prophylaxis before treatment, those who had treatment without prophylaxis and those yet to initiate PMTCT showed 32% (8), 5% (3) and 15% (4) HIV-1 drug resistance associated mutations respectively. The differences were significant with p value < 0.05. Resistance Associated Mutations (RAMs) were seen in 14 participants (35%) to nucleoside reverse transcriptase inhibitors (NRTIs) and non-nucleoside reverse transcriptase inhibitors (NNRTIs). The most common NRTI mutation found was M184 V; K103 N and A98G were the most common NNRTI mutations seen. Thymidine Analogue Mutations (TAMs) such as M41 L, K70R and T215Y were found in all the groups; the most common of the TAMs found were M41 L and T215Y. Majority of the subtypes were CRF02_AG (82%).

**Conclusion:**

In Ghana initiation of uninterrupted treatment upon diagnosis, coupled with drug resistance testing, would produce a better treatment outcome for HIV-1 positive pregnant women.

## Background

Antiretroviral therapy (ART) was started in Ghana in 2003 and has gone through a number of revisions to provide appropriate health care and support for HIV positive persons across the whole of Ghana [1]. The National AIDS/STI Control Programme (NACP) of the Ghana Health Service implemented various research-backed interventions to monitor drug resistance known to arise in HIV patients through the use of the Antiretrovirals (ARVs) [2]. The emergence of HIV-1 drug resistance viral strains is a major obstacle in the effective management of HIV infection and AIDS. Drug resistant strains may develop due to exposure to drugs but drug naïve persons could also be infected with drug-resistant strains [3]. The Ghana HIV Drug Resistance (HIVDR) Threshold Survey was initiated in 2007 [4] to generate information on the presence of HIV drug-resistant strains in the locality where Ghana’s ART for HIV was first introduced. It was also to seek information on active transmission of HIV drug-resistant strains in drug-naïve persons in the country so as to signal action to address transmitted HIV drug resistance (HIVDR) in Ghana. A Survey of Emergence of HIV Drug Resistance was also initiated by the NACP to monitor the emergence of HIV drug resistance in Ghana amongst patients initiating antiretroviral therapy (ART). These two surveys were designed to monitor the impact of HIV-1 drug resistance on the ART programme [4].

However, a group of patients fall in a grey area not covered directly by the two surveys. This group comprised HIV positive women given antiretrovirals as prophylaxis to prevent the transmission of HIV to their babies during pregnancy and subsequently given treatment for their own health post-partum. Bearing in mind the emergence of drug resistance in the face of antiretroviral pressure, these women were given the same ARVs in the treatment phase as they were given during the prophylaxis or the phase of preventing the transmission of the infection from the mother to the infant [2]. The effectiveness of the ARVs with such a background was becoming questionable in the absence of data on the resistance profiles of these women.

This study was thus designed to determine HIV-1 drug resistance mutations present in Ghanaian women, to characterize any resistance mutations found according to the class of antiretrovirals (ARVs) used during treatment and to provide data on the profile of HIV-1 drug resistance present in Ghanaian women on treatment.

## Methods

### Study design and sites

This was a cross-sectional study carried out between 1st November, 2010 and 30th November, 2011 and used the convenient sampling technique to enroll 116 HIV-1 positive Ghanaian women who accessed care and support at seven National AIDS/STI Control Programme (NACP) centres in three regions of Ghana.

### Study participants

The study involved two groups of HIV-infected mothers and a group of HIV positive pregnant women at gestational periods less than 28 weeks. One group of mothers (Group 1) was made up of HIV-positive mothers who had been on antiretroviral prophylaxis for prevention of transmission of the virus to the foetus when they were pregnant and had subsequently been put on full ART for their own health needs post-partum (Prophylaxis plus ART Group). The second group (Group 2) comprised HIV-positive pregnant women who had not had any prior exposure to ARVs at the time of the study (Drug-Naïve) and were pregnant at less than 28 weeks. A third group (Group 3) was made up of mothers who had initiated ART when they were pregnant without prophylaxis (Drug-experienced without prophylaxis) because their CD4 count levels were below 350 cells /μL at the time of ARVs initiation.

During the study period, the PMTCT programme in Ghana administered a combination of Zidovudine (AZT) and Lamivudine (3TC), both NRTIs, (known as Combivir) to the patients from 28 weeks of pregnancy as prophylaxis until labour onset when a single dose Nevirapine (sd NVP), an NNRTI, was added. In post-PMTCT periods when these mothers needed ARVs for their own health, they were given the same drugs as during the prophylaxis phase- 3TC, AZT and NVP or Efavirenz (EFV-another NNRTI) but this time as a triple therapy. Didanosine (DDI) or Abacavir (ABC)–both NRTIs, or Tenofovir (TDF), a Nucleotide Reverse Transcriptase Inhibitor (NtRTI) substituted AZT for some of the participants. HIV-positive pregnant women who reported at the Care and Support Centres and had CD4 levels below 350cells/μL were given the triple therapy without a prophylactic phase [2].

### Sample Collection & Processing

A structured questionnaire was used to obtain basic socio-demographic and clinical data from cases and controls. Upon explaining the study and obtaining written informed consent from the patients at the study sites, whole blood sample was taken from the antecubital vein of each participant into Ethylenediamine tetra-acetic acid (EDTA) treated tubes. The samples were placed in an ice chest with frozen ice packs and transported to the Virology Department of Noguchi Memorial Institute for Medical Research (NMIMR) at Legon, Accra, Ghana, where the plasma was separated from the whole blood through centrifugation at 2000 *g* for 10 min and stored at minus (−) 70 °C until analyzed.

### HIV-1 drug-resistance genotyping

Viral RNA was extracted from 140 μL of plasma samples using QIAamp viral RNA mini kit (Qiagen, USA). QIAGEN One-Step RT-PCR Kit was used for the amplification, according to the manufacturer’s protocol [5]. Primers used for the RT gene and the PR gene in the Round 1 amplification were DRRT1L/DRRT4L and DRPRO5/DRPRO2L respectively, as has previously been described [6]. The thermal cycling conditions applied were described previously [7].

Further amplification of the round 1 products was done by nested PCR using AmpliTaq Gold Master Mix Kit (ABI, USA) with primers DRRT7L/DRRT6L and DRPRO1M/DRPRO6 for RT and PR genes respectively, and the thermal cycling conditions used were as previously described [6, 7].

The products of the RT and PR gene from the nested PCR assay were verified through the use of agarose gel electrophoresis and the bands in the gel were visualized using a Gel Documentation system (GEL-LOGIC 100 Imaging System from Kodak) with a High Performance Ultraviolet-Transilluminator (UVP, UK). The amplicons were purified using QIAquick PCR Purification Kit (QIAGEN, USA) to obtain the DNA products needed for sequencing, following the manufacturer’s instructions. Cycle sequencing was performed on the purified PCR products using the Big Dye Terminator Cycle Sequencing Kit vs 3.1 from Applied Biosystems Inc. (ABI), USA. An ABI 2720 thermal cycler was used with the following conditions 94 °C 2 min/ (94 °C 30s; 50 °C 15 s; 60 °C 4 min) for 25 cycles/4 ^o^ C hold. The Primers and conditions used at this stage were as previously described by Villahermosa et al. [7] and Fujisaki et al. [6].

The DNA products obtained were purified using the CentriSep Column Purification Method (Princeton Separations, Inc., Adelphia, NJ, USA) by following the manufacturer’s instructions. The purified DNA samples were loaded into the ABI Genetic Analyzer 3130 (Applied Biosystems, USA) for the automatic analysis of the HIV-1 sequences generated.

Sequences were edited using the Align IR version 2.0 software (from LI-COR Inc., Michigan Technology University, 2001). The consensus sequences in their FASTA format were submitted online to the Stanford University HIV Database Programme (http://hivdb.stanford.edu) to generat the resistance data and to assign subtypes for each sample as well as for the interpretation of any resistance data elicited. The resistance associated mutations were considered for analysis taking cognizance of the 2013 IAS-USA recognized mutations for NRTIs, NNRTIs and PIs [8].

### HIV-1 subtyping by phylogenetic analysis

The edited sequences were submitted to the GenBank database using nucleotide-nucleotide BLAST search programme (BLASTN 2.2.29+) of the NCBI website [9, 10]. Sequences homologous to the study sequences were retrieved from the DNA databanks for comparisons. The sequence data were aligned using the CLUSTAL W package [11] integrated into the Bioedit 7.25 software suite [12].

**Fig. 1:**
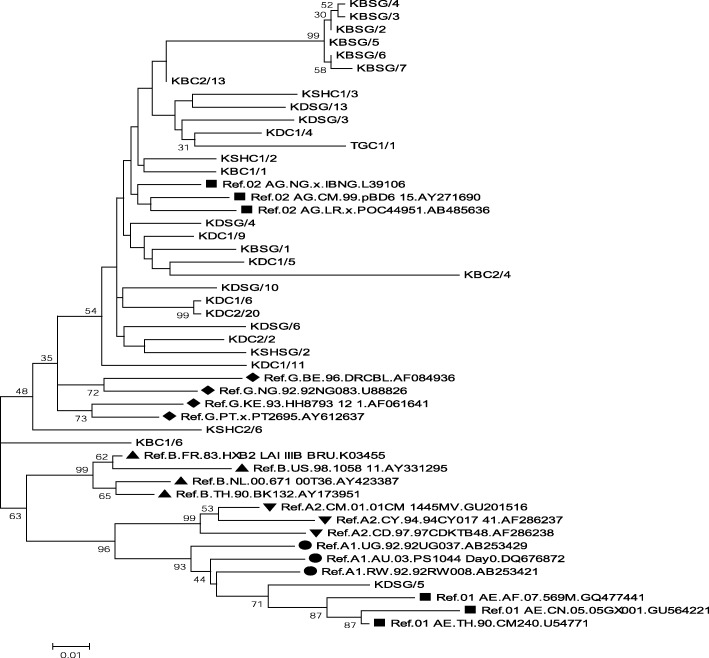
Molecular Phylogenetic Analyses for RT sequences and selected HIV-1 subtype references by maximum likelihood method. Markers indicate reference sequences: ▲ Ref B, ▼ Ref A2, ● Ref A1, ♦ Ref G, ■ Circulating recombinant forms (CRFs)

In generating the phylogenetic tree the evolutionary history was inferred by using the Maximum Likelihood method based on the Tamura-Nei model. The percentage of trees in which the associated taxa clustered together is shown next to the branches (see Fig. 1). Initial tree(s) for the heuristic search were obtained by applying the Neighbor-Joining method to a matrix of pairwise distances estimated using the Maximum Composite Likelihood (MCL) approach. The tree was drawn to scale, with branch lengths measured in the number of substitutions per site. The analysis involved 49 nucleotide sequences. All positions containing gaps and missing data were eliminated. There was a total of 533 positions in the final dataset. The evolutionary analyses were conducted in MEGA 6 [13].

### Statistical analysis

Descriptive analysis was done, with percentages comparing proportions of relevant variables. Stanford HIV Database programme was used for drug resistance analysis, interpretation and subtyping with phylogenetic analysis supporting the classifications into subtypes. All the data was entered into an Excel database and then exported into SPSS version 16.0 software (SPSS Inc., Chicago, Illinois) for the statistical analysis, using *p* values to compare the significance between the different groups and the emergence of drug resistance mutations.

## Results

### Characteristics of study participants

Out of 116 participants, 25 (21.6%) were mothers who were on ART after previous PMTCT prophylaxis (Group 1- Prophylaxis plus ART group), 26 (22.4%) were pregnant HIV-positive drug-naïve participants (Group 2- Drug-Naïve group), and 65 (56.0%) were mothers who had been put directly on ART without prophylaxis as a result of low CD4 + T cell count at the time they were pregnant (Group 3- Drug-Experienced without Prophylaxis group).

The age range of the participants was 20 to 46 years with the mean ages (in years) being 33.1(±5.7), 30.7(±5.6) and 33.4(±4.7) for Group 1, Group 2 and Group 3 respectively [28].

The study participants were at different stages of disease progression according to the WHO Clinical Staging method. For Group 1 participants (Prophylaxis plus ART group), 44% were at Stage I, 52% at Stage II, 4% at Stage III and none at Stage IV of the infection. Of the participants in Group 2 (drug-naïve group), 57.7% were at Stage I and 42.3% were at Stage II of the HIV infection with no one at Stages III and IV. With the mothers in Group 3 (drug-experienced without prophylaxis group), 18.5% were at Stage I of the infection, 36.9% at Stage II, 41.5% at Stage III and 3.1% at Stage IV of the infection.

### Emergence of HIV-1 drug resistance associated mutations

The study detected drug resistance associated mutations (DRAMs) in participants from each group. Thirteen percent (15/116) of samples showed the presence of drug resistance associated mutations (DRAMs); out of 40 sequences obtained for the RT gene, 35%, (14/40) showed resistance associated mutations with the reverse transcriptase gene for both NRTIs and NNRTIs. Out of the 33 sequences successfully obtained for the PR gene, 3% (1/33) had resistance associated mutations in the protease gene for Protease Inhibitors.

Amongst the participants in the drug-naïve group 15% (4/26) had resistance associated mutations, whilst in the Prophylaxis plus ART group 32% (8/25) of participants showed resistance associated mutations and participants in the drug-experienced without prophylaxis group 5% (3/65) showed DRAMs (Table 1). The difference among the groups and the emergence of DRAMs was significant, *p* < 0.05.

**Table 1:** Association between the study variables and the emergence of HIV-1 drug resistance

Study variables		HIV drug resistance			*P* value
		DRAMs, N (%)	No DRAMs, N (%)	Total N (%)	
Study groups	1 (ART with prophylaxis)	8(32)	17(68)	25(21.6)	.002^a^
	2 (Drug-Naïve)	4(15)	22(85)	26(22.4)	
	3 (ART with no prophylaxis)	3(5)	62(95)	65(56.0)	
Duration on art (Groups 1 & 3)	< 1 Year	4(16.7)	20(83.3)	24(26.7)	.624^a^
	1-2 Years	5(12.5)	35(87.5)	40(44.4)	
	≥ 3 Years	2(7.7)	24(92.3)	26(28.9)	
Who clinical staging	1	9(23.7)	29(76.3)	38(32.8)	.113^a^
	2	4(8.3)	44(91.7)	48(41.4)	
	3	2(7.1)	26(92.9)	28(24.1)	
	4	0(0)	2(100)	2(1.7)	
Adherence	Yes	10 (12.0)	73 (88.0)	83(92.2)	.560^a^
	No	1 (14.3)	6 (85.7)	7(7.8)	

^a^ *Significant at 5%*

The presence of DRAMs seen in the study as a result of the association between the participant’s duration on ART, WHO clinical staging and adherence to treatment, and the emergence of such DRAMs are shown in Table 1; the difference between these parameters and DRAMs were not significant, *p* > 0.05.

Major drug resistance associated mutations (DRAMs) to both the NRTIs and the NNRTIs were seen in this study, as shown in Table 2 for the three different groups in the study.

**Table 2:** HIV-1 Drug Resistance Associated Mutations (DRAMs) in the study participants

Participant groups	Reverse transcriptase DRAMs	
	NRTIs	NNRTIs
Art after prophylaxis (Group 1)	M184 V	K103 N,Y181C
	M41 L,M184 V, T215Y	K103 N,M230 L,A98G,
	M41 L,M184 V,T215Y	K103 N A98G,
	M41 L,M184 V,T215Y	A98G, K103 N
	M41 L,M184MV,T215Y	A98G,K103 N,M230 LM
	M41 L,M184MV,T215Y	A98G,K103 N,M230 LM
	M41 L,L74 V,T215Y	K103 N, A98G,L100IL,,M230 L
	K219KR	G190EG
Drug naïve group (Group 2)	V75S	E138A
	NONE	A98G
	M184 V	K103 N
	L210 W	V106A
Art without prophylaxiS (Group 3)	M184 V,Y115F,T215S	A98G
	M41 LM,D67G,K70R,K219E,T215I,M184 V	A98G
	M184 V	NONE

*DRAMS Drug Resistance-Associated Mutations to Reverse Transcriptase Inhibitors*

For Group 1 (Prophylaxis plus ART Group) all the participants were given the same combination of ARVs as prophylaxis (AZT, 3TC and NVP) with the exception of one patient who was given only NVP as prophylaxis. The subsequent ART regimen for all Group 1 mothers was the same. There was no drug resistant associated mutation (DRAM) to Protease Inhibitors (PIs) in this group. The major drug resistance associated mutations to NRTIs seen among the Group 1 participants were M41 L, M184 V, M184MV, L74 V and T215Y with no minor drug resistant associated mutations to NRTIs in this group. The most commonly seen drug resistant associated mutations to NRTIs in this group were M184 V, T215Y and M41 L.

Major DRAMs to NNRTIs seen in the Prophylaxis plus ART Group (Group 1) were K103 N, Y181C, M230 L and L100IL and the minor DRAMs to NNRTIs seen was A98G. The most common HIV-1 drug resistance associated mutations seen with the NNRTIs were K103 N, M230 L and A98G. There were no resistance associated mutations with regards to the Protease Inhibitors (PIs) and no participants in this Group had been treated with a Protease Inhibitor.

In the drug-naïve participants group (Group 2), there were no drug resistance associated mutations with Protease Inhibitors either. However, there were four (4) participants (15%) showing DRAMs to NRTIs and NNRTIs. Two major HIV-1 drug resistance associated mutations for NRTIs, M184 V and L210 W, were seen in two of the participants with one minor DRAMs to NRTIs, V75S, seen in one patient; one patient did not have any drug resistance mutation to NRTIs. Three major DRAMs to NNRTIs were seen in 3 patients among the drug-naïve participants; these DRAMs were K103 N, V106A and E138A. One minor drug resistance associated mutation, A98G, was seen in one patient in this group [28].

In Group 3 where participants had received ART but no Prophylaxis (drug-experienced without prophylaxis group) for prevention of mother-to-child transmission (PMTCT) of HIV, 3 patients (5%) showed drug resistance associated mutations to NRTIs, NNRTIs and PIs. Major and minor DRAMs to NRTIs, NNRTIs and PIs were seen in one patient who had been given NRTIs and a PI (Nelfinavir) initially, followed by other NRTIs and an NNRTI. The major DRAMs to NRTIs seen in this group were M184 V, Y115F, K70R, K219E and M41 LM while the minor DRAMs to NRTIs seen were T215S, T215I and D67G. There were no major DRAMs to NNRTIs in this group though the minor drug resistance mutation, A98G, was seen in two of the patients. The major DRAMs to PIs seen in the group was I84V; the minor drug resistance mutations seen were A71V, L89 V and M46MV. M184 V mutation was found to be the most common mutation among this group of mothers.

Thymidine analogue mutations (TAMs) were seen in this study; these were M41 L, K70R, L210 W, T215Y and K219E. The most common TAM seen in this study were M41 L and T215Y. These appeared in 7 (88%) out of the 8 participants with DRAMs in Group 1 which had prophylaxis followed by treatment.

#### Distribution of HIV-1 subtypes

All the sequences obtained were subtyped using the Stanford HIV database drug resistance programme (www.hivdb.stanford.edu), which produced 33 (82%) CRF02_AG subtypes, 2 (5%) subtype CRF01_AE, 1 (3%) subtype A, 2 (5%) subtype B and 2 (5%) subtype G for the RT gene whilst the PR gene had 32 samples (97%) being subtype CRF02_AG and 1(3ó%) subtype A. Subtyping by phylogenetic analysis was also performed though some of the sequences for the RT gene were excluded from the phylogenetic tree-building (Fig. 1) because they were relatively shorter fragments. The Phylogenetic analyses were conducted in MEGA 6. Majority of study sequences clustered with the circulating recombinant form CRF02_AG.

Of the 8 mothers in Group 1(Prophylaxis plus ART) with HIV-1 drug resistance associated mutations, 7 (88%) were of subtype CRF02_AG and 1(13%) was of CRF01_AE. Out of the 4 mothers (15%) in Group 2 (Drug-Naïve patients) with HIV-1 drug resistance associated mutations, 3 (75%) were of subtype CRF02_AG and 1 (25%) was of subtype A. Two of the HIV-1 strains showing drug resistance associated mutations in Group 3 were of Subtype CRF02_AG and one was of Subtype B.

## Discussion

This study showed that there is a significant association between the emergence of HIV-1 drug resistance and the various groups of HIV-1 positive participants, *p* value < 0.05 (Table 1). Under the PMTCT programme, the mothers who were on ART after previous prophylaxis showed the most drug resistance associated mutations (32% DRAMs out of the total in Group One). Mothers who were put on ART directly without ARV prophylaxis showed the least level of HIV-1 drug resistance (5%), an indication of a better outcome for patients of this category. The difference in the proportions was significant with p value being 0.002 (Table 1).

Participants in Group 3 (ART without prophylaxis) had been on ART for a longer time than participants in Group 1- a mean of 34 months duration in the former compared to 11 months in the latter group (Table 1). However, there was no significant difference (*p* value > 0.05-Table 1) among the participating groups with regards to the impact of duration on ART on the emergence of HIV-1 DRAMs. The longer a patient is on the HIV-1 drug does not necessarily lead to higher resistance outcomes. Initiating ART upon HIV diagnosis without prior prophylaxis had a better outcome in preventing the emergence of HIV-1 drug resistance. The study thus provided data to support the adoption of the new Option B Plus proposed by the WHO for all HIV-1 infected pregnant Ghanaian women regardless of the CD4 counts [14, 15]. This is also in consonance with the new WHO guidelines on when to start treatment [16]. This study has shown that the target of eliminating mother-to-child transmission of HIV-1 in Ghana would be enhanced by giving ART upon diagnosis without prophylaxis. The study also provided initial evidence for Ghana that addressed concerns about long term use of ART when initiated in early HIV infection and the emergence of HIV-1 drug resistance. In Table 1, participants who had been longest on ART (≥3 years) presented the least level of HIV-1 drug resistance as compared to those who had been on ART for lesser periods (1 to 2 years). Adherence to treatment was a contributory factor in this situation as shown by high adherence levels achieved in this study.

Of the drug-naïve group in this study, 4 (15%) of the participants showed the presence of HIV-1 drug resistance associated mutations. Some of these mutations reduce the susceptibility of NRTIs and others reduce the effect of NNRTIs [8]. The drug-naïve Ghanaian women in this group were found to be harbouring strains of the virus resistant to the drug regimen available to them even before initiating PMTCT. Hence the effect of the drugs was suboptimal. Though drug resistant strains may develop due to exposure to drugs, HIV positive persons could be infected with drug-resistant strains or pre-existent resistant strains [3]. The implication of the pre-existent resistant strains encountered in the drug-naïve group is the lack of effective drug options for treatment. Thus there would be the need to switch them from the first line regimen they were on to a second line regimen option. Without drug resistance testing prior to treatment initiation these people would continue to post suboptimal responses to the treatment. For these people NNRTI in use in Ghana, Nevirapine and Efavirenz would not be effective so Etravirine and Rilpivirine would have to be introduced by the policy makers.

This study further re-emphasized the need to carry out genotypic resistance testing for pregnant HIV-1 positive women before initiating PMTCT in Ghana. Drug resistance could occur when resistance mutations emerge because of drug-selective pressure in individuals receiving antiretroviral therapy [3]. This study has shown that HIV-1 drug resistant associated mutations had emerged in mothers who had received ART for their own health after previous exposure as prophylaxis to prevent the transmission of HIV-1 to the baby. The HIV-1 drug resistance associated mutations (DRAMs) encountered in the study have different effects on the susceptibility of the ART administered to the patients enrolled in the study groups.

### ART with prophylaxis group

For the mothers who were on ART after prophylaxis in the PMTCT programme, the HIV-1 drug resistance associated mutations seen were dominated by M184 V for NRTIs and Thymidine Analogue-Associated Mutations (TAMS) including M41 L and T215Y; and K103 N with A98G for NNRTIs. Generally, it is known that mutations selected by TAMS confer resistance to internationally approved NRTIs; examples of such TAMS encountered in the study are M41 L, D67N, K70R, L210 W, T215Y/F and K219Q/E [8]. TAMs which confer resistance to nucleoside analogues were seen among the DRAMs in all the 3 different groups.

It is known that mutations selected by TAMS, as seen in this Group, confer reduced susceptibility to the currently approved NRTIs used in Ghana [17]. The study participants had been previously given a combination of Zidovudine (AZT) and Lamivudine (3TC) which are both NRTIs with Nevirapine (NVP) or Efavirenz (EFV) (both NNRTIs). Though none of the mothers had been given ABC, TDF, DDI or D4T, resistance to these ARVs had emerged in these participants. This could be due to the effect of cross-resistance in the drug classes [3, 18].

HIV-1 drug resistance mutations to NNRTIs seen in this study all work against the susceptibility of HIV to the ARVs recommended for use in Ghana. K103 N was seen in all the participants in the Prophylaxis plus ART Group (Group 1) and caused high-level resistance to NVP and EFV. The two NNRTIs are widely used in Ghana. When K103 N is seen in combination with L100I as seen in this group, it confers high-level resistance to both NVP and EFV, leaving the patient with only the AZT and 3TC combination to counter the virus. Furthermore, A98G found in the group causes reduction in susceptibility to NVP by 5-fold and to EFV by 3-fold and has the ability to cause reduction in other members of the NNRTIs not in use in Ghana such as Etravirine (ETR) and Rilpivirine (RPV). NNRTIs resistance mutations Y181C and M230 L are known to confer high-level and intermediate resistance to NVP and EFV [8]. These were also found to be present in the participants in the Prophylaxis plus ART Group (Group 1). The presence of Y181C, M230 L and M230 LM posed resistance to all the NNRTIs used in the country for this group of people (the prophylaxis plus ART group). The interplay of these resistance mutations restricts the options available for such patients (Table 3).

**Table 3:** Implications of Drug Resistance Associated Mutations Detected

HIV-1 Drug status	Mutations	Drug resistance	Useful drug options
NRTI			
Art after prophylaxis	M184 V	3TC,FTC,DDI,ABC	AZT,TDF D4T
	M184 V,M41 L,T215Y	ABC,AZT,D4T,DDI, TDF,3TC,FTC	NONE
	M184 V,M41 L,L74 V, T215Y	ABC,AZT,DDI,	3TC,FTC
Art no prophylaxis	M184 V,D67G,M41 LM, K70R,K219E,T215I	3TC,AZT,FTC,ABC, D4T,DDI,TDF	NONE
	M184 V	3TC,FTC,DDI,ABC	AZT,TDF,D4T,
	M184 V,Y115F,T215S	ABC,TDF	3TC,FTC,DDI
Drug-naive	M184 V	3TC,ABC,DDI,FTC	AZT,D4T,TDF
	L210 W	AZT,ABC,DDI,FTC, TDF	3TC
NNRTI			
Art after prophylaxis	K103 N,Y181C	NVP,EFV,ETR,RPV	NONE
	A98G,K103 N,M230 LM	NVP,EFV,ETR,RPV	NONE
	K103 N,M230 L,L100IL, A98G	NVP,EFV,ETR,RPV	NONE
Art no prophylaxis	A98G	NVP	EFV
Drug-naive	K103 N	NVP,EFV	NONE
	V106A	NVP,EFV	NONE
	E138A	NVP,EFV,ETR,RPV	NONE
	A98G	NVP	EFV

*NRTI Nucleoside Reverse Transcriptase Inhibitors, NNRTI Non-Nucleoside Reverse Transcriptase Inhibitors*

### Drug-experienced without prophylaxis group

HIV-1 drug resistance seen in the group of participants who had been given ART without prophylaxis (drug-experienced without prophylaxis) was only 5% (3 out of 65 participants). The resistance associated mutation in the RT gene to NNRTIs (NVP & EFV) seen in this group was A98G; this was found in two of the participants who had been on treatment for almost 6 years. No mutation with resistance to NNRTIs was seen with the third participant, KDC2/20, who had been on the ARVs for only 10 months. The presence of A98G in these mothers could confer high-level resistance to NVP and EFV [19]. Resistance mutation to NRTIs seen in the group of drug-experienced without prophylaxis mothers was M184 V. M184 V as a stand-alone mutation, results in a clinically significant reduction in HIV-1 replication in the patient [20]. This situation was seen with participant KDC2/20 who had been on treatment for less than 12 months as at study time and had achieved viral suppression (undetectable level by the assay). These findings support the recommendation by WHO in the Drug Resistance Report of 2012 [21] that virological testing should be carried out at 12 months after initiating treatment, as an additional early warning indicator for better prevention of emergence of drug resistance mutations. However, in the other mothers in this group, M184 V occurred with Thymidine Analogue-Associated Mutations (K70R, K219E, M41 LM and T215I); it therefore produced a synergistic effect that led to different levels of reduction in susceptibility [3, 8]. The presence of M184 V with TAMs confer resistance to all the ARVs available to the patient, resulting in no useful option in NRTIs the this category of patients (Table 3).

One of the participants in this group, KBC2/13, had initiated treatment with Combivir (a combination of AZT and 3TC) together with Nelfinavir (NFV), a Protease Inhibitor (PI). Mutation I84V emerged in the Protease gene associated with resistance to the PIs recommended for use in Ghana [22], ie NFV and Ritonavir boosted Lopinavir (LPV/r). This is a major resistance mutation to PIs and confers intermediate- to high-level resistance to NFV and LPV/r [8].

### Drug-Naïve pregnant group

Mutations associated with NRTIs and NNRTIs emerged in 4 participants in the drug-naïve group of the study (Table 2). One patient (KDC1/6) had M184 V mutation which is a major mutation in the RT gene associated with NRTIs and known to confer high-level resistance to 3TC and FTC, low-level resistance to DDI and ABC. The V75S mutation is weakly selected by NRTIs and thus confers a low-level resistance to DDI and D4T.

L210 W is a TAM, a major mutation detected in one participant in this group (KDC1/10); it confers low-level resistance to all NRTIs in use in Ghana except 3TC and FTC.

K103 N and V106A are major mutations associated with NNRTIs conferring high-level HIV-1 drug resistance to NVP and EFV. A98G is a minor mutation found in one drug-naïve participant (KDC1/1) and causes 5fold and 3fold reduced susceptibility to NVP and EFV respectively. Though both patients KDC1/6 and KDC1/10 were HIV-1 drug-naïve, they had major resistance mutations for both NRTI and NNRTI [8, 28]. The options of ARVs open to this group of HIV-1 positive persons in Ghana is seriously limited even before treatment is initiated (Table 3).

E138A mutation in the RT gene was seen in this group and though it does not cause reduction in susceptibility to NVP and EFV, it confers low-level resistance to RPV and ETR, other NNRTIs not used in Ghana, pointing to another case of cross-resistance.

No major mutation in the Protease gene associated with PIs for the drug-naïve group was seen in this study.

The HIV-1 strains in this study were predominantly subtype CRF02_AG of HIV-1, confirming the findings of other studies in Ghana that HIV-1 CRF02_AG is the prevalent subtype in the country [24, 25]. The diversity of the circulating subtypes of HIV-1 strains seen in this study was assessed using both the Stanford HIV database programme and via phylogenetic analysis (Fig. 1).

The subtype CRF02_AG (Ghana) seen in this study was found to be related closely to CRF02_AG from Nigeria, Cameroun and Liberia (Fig. 1) confirming strain related mutations and the genetic complexity of HIV-1 infection in the west coast of Africa as shown by other studies [23, 26].

## Conclusions

In summary, the proportion of patients with HIV-1 drug resistance mutations was found to be significantly higher in the mothers with a history of prophylaxis before initiation of treatment compared to mothers who initiated treatment without prophylaxis. Subsequently the study determined that mothers who previously had prophylaxis and were on ART were more likely to develop drug resistance mutations than those on ART without prior prophylaxis. Thus, in Ghana initiation of uninterrupted treatment upon diagnosis coupled with drug resistance testing would help to produce a better treatment outcome for Ghanaian HIV-1 positive mothers and pregnant women.

This endorsed the initiation of treatment upon diagnosis for all HIV positive pregnant Ghanaian women on ART irrespective of the level of their CD4 counts, in consonance with WHO recommendation for treating HIV in pregnant women currently with the recommended ARVs [27].

### Study limitations

The study encountered difficulties in recruiting HIV positive mothers at the study sites since contact telephone numbers provided in the patient hospital folders were mainly unreachable and in some cases incorrect. This posed a limitation to the sample size due to the number of defaulting patients who, however, met the study criteria. The non-inclusion of drug resistance testing nationally was a limitation to the study since it would have added to the understanding of the cause of the DRAMs for drug-naïve HIV positive persons.

## Abbreviations

3TC: Lamivudine
ABC: Abacavir
AIDS: Acquired immune deficiency syndrome
ART: Antiretroviral therapy
ARVs: Antiretrovirals (antiretroviral drugs)
CD4: Cluster of differentiation type 4
D4T: Stavudine
DDI: Didanosine
EFV: Efavirenz
ETR: Etravirine
FTC: Emtricitabine
HIV: Human immunodeficiency virus
IAS: International aids society
NFV: Nelfinavir
NMIMR: Noguchi memorial institute for medical research
NNRTIs: Non-nucleoside reverse transcriptase inhibitors
NRTIs: Nucleoside reverse transcriptase inhibitors
NtRTIs: Nucleotide reverse transcriptase inhibitors
PIs: Protease inhibitors
TDF: Tenofovir
WHO: World health organization
ZDV: ZIDOVUDINE
